# How music-induced emotions affect sexual attraction: evolutionary implications

**DOI:** 10.3389/fpsyg.2024.1269820

**Published:** 2024-04-10

**Authors:** Manuela M. Marin, Bruno Gingras

**Affiliations:** ^1^Department of Cognition, Emotion and Methods in Psychology, University of Vienna, Vienna, Austria; ^2^Austrian Research Institute of Empirical Aesthetics, Innsbruck, Austria; ^3^Department of Cognitive Biology, Faculty of Life Sciences, University of Vienna, Vienna, Austria

**Keywords:** origins of music, mate choice, sex appeal, sexual selection, arousal transfer, social bonding, evolution of musicality, dating

## Abstract

More than a century ago, Darwin proposed a putative role for music in sexual attraction (i.e., sex appeal), a hypothesis that has recently gained traction in the field of music psychology. In his writings, Darwin particularly emphasized the charming aspects of music. Across a broad range of cultures, music has a profound impact on humans’ feelings, thoughts and behavior. Human mate choice is determined by the interplay of several factors. A number of studies have shown that music and musicality (i.e., the ability to produce and enjoy music) exert a positive influence on the evaluation of potential sexual partners. Here, we critically review the latest empirical literature on how and why music and musicality affect sexual attraction by considering the role of music-induced emotion and arousal in listeners as well as other socio-biological mechanisms. Following a short overview of current theories about the origins of musicality, we present studies that examine the impact of music and musicality on sexual attraction in different social settings. We differentiate between emotion-based influences related to the subjective experience of music as sound and effects associated with perceived musical ability or creativity in a potential partner. By integrating studies using various behavioral methods, we link current research strands that investigate how music influences sexual attraction and suggest promising avenues for future research.

## 1 Introduction

Music, besides language, is one of the main distinctive characteristics of the human species (Benítez-Burraco and Nikolsky, 2023). Whereas language is primarily a means to communicate semantic meaning, music is a powerful elicitor of emotions (e.g., Juslin and Sloboda, 2011; Marin and Bhattacharya, 2011). Music fulfills a myriad of functions in our daily lives (Clayton, 2016) that go beyond pure entertainment and music-induced pleasure (Pinker, 1997). Abundant empirical evidence for a socio-biological basis of music has accumulated over the recent years, which suggests that music may have evolved through sexual and/or natural selection (Huron, 2001; Wallin et al., 2001; Honing and Ploeger, 2012), though the exact selection mechanism has been a longstanding topic of debate (Kleinman, 2015). In this review, we present evidence suggesting that music may have evolved through sexual selection as proposed by Darwin (1871). In our discussion on the putative role of music in mate choice, particular emphasis will be placed on underlying psychological mechanisms based on music-induced emotions in listeners.

## 2 Origins of musicality

One difficulty encountered when studying the origins of music is that, although music is a phenomenon observable in most, if not all, cultures (Blacking, 1973), it turns out to be difficult to define according to universally observable characteristics such as rhythm or pitch (Savage et al., 2015). Some musical genres have no underlying isochronous pulse (e.g., Tibetan monotone chanting, Bispham, 2009), whereas others do not make use of discrete pitches (e.g., some compositions by Xenakis). Thus, from an evolutionary perspective, it may prove more useful to study the origins of *musicality*, defined as the set of traits associated with the capacity to produce and enjoy *music*, defined as the cultural end-product of musicality (Honing et al., 2015).

Another difficulty is that musical practice leaves only few artifacts such as recordings or fossilized vocal organs (Killin, 2018). Consequently, accounts of the origins of music remain in large part interpretative: scientists attempt to reconstitute the past based on scant pre-historical evidence, such as bone flutes that are at least 35,000 years old in *Homo Sapiens* (Conard et al., 2009) and Neanderthals (Turk et al., 2020), but musical behavior such as singing, drumming and dancing cannot be traced back to fossils. One could tentatively speculate, based on the existence of these bone flutes, that the common ancestor of both species was to some extent already musical 631-789 k years ago (Beerli and Edwards, 2002). However, both species were interbreeding for a while in Europe (Nigst et al., 2014; Vidal-Cordasco et al., 2023), which weakens such speculations. The current archeological evidence is augmented by complementary comparative evidence such as cross-cultural studies, which look for common features or universals (Brown and Jordania, 2013), or cross-species comparisons, which are used to draw inferences about ancestral forms and putative adaptive functions associated with musicality.

Among the current accounts of the origins of music, a broad dichotomy can first be observed between adaptationist and non-adaptationist theories. Whereas the former propose that musicality, as a trait, plays a role in the survival of the human species, the latter consider music to be either a “technology” or a byproduct of other adaptations which plays no evolutionary role (Pinker, 1997). Adaptationist accounts can be further subdivided according to the principal function they ascribe to musicality, or to the mode of selection (natural versus sexual). Sexual selection itself comprises two pathways, namely intrasexual competition, involving dominance and prestige (Fisher and Candea, 2012; Varella et al., 2017), and intersexual selection, associated with fitness indicators as well as aesthetic aspects (see Prum, 2012; Davis and Arnocky, 2022). Modern sexual selection theory discusses several intersexual processes, such as good taste (Davis and Arnocky, 2022), good genes, good partners, and good providers (Buss and Shackelford, 2008), good parents (Kreutz and Feldhaus, 2023) and good relationship maintenance (Evans et al., 2022). Crucially, adaptationist accounts are not necessarily mutually exclusive: although various theories emphasize distinct, and possibly complementary, functions for musicality, they broadly agree that evolutionary pressures played a defining role in the origins of musicality.

In aesthetics, one frequently discussed account is Darwin’s sexual selection hypothesis (Darwin, 1871; Prum, 2012; Renoult, 2016; Kalinowski et al., 2021), according to which music acts as a courtship display in reproductive mate choice. Darwin’s hypothesis, which states that trait-preference covariation may occur without necessarily having a biological function, was revived by Miller (1999, 2000, 2001), who emphasizes the fitness indicator role of the sexually selected trait (see also Sluming and Manning, 2000) and regards music as an honest signal. Supporting this hypothesis, Miller noticed that interest for music peaks in adolescence and that young male musicians produce more music than female musicians (see also Savage et al., 2015). Musicality was also reported to be a preferred trait in romantic partners (Kaufman et al., 2016).

Another longstanding theory is based on the idea that music and language have a common origin (Darwin, 1871), with language subsequently specializing in the communication of semantic meaning, and music primarily conveying emotions (Ma et al., 2019). This account, recently revisited by researchers such as Brown (2000a) and Mithen (1998), has been strengthened by neuroimaging evidence showing that music and language processing share neural resources (Koelsch et al., 2004; Patel, 2008). Language itself may be a product of sexual selection (Worden, 2022), and since both communication systems may have co-evolved (Benítez-Burraco and Nikolsky, 2023), positive effects of music on sexual attraction are probably not a recent phenomenon. Other theories suggest a role for music in promoting group cohesion (Dunbar, 2004; Savage et al., 2021), mood-regulation (Sloboda and O'Neill, 2001), mother-infant bonding (Trehub, 2003; Dissanayake, 2008; Mehr et al., 2021), territorial defense (Hagen and Hammerstein, 2009), or cognitive and social development (Cross, 2001). Future studies should aim to show which aspects of musicality can solidly be ascribed to either natural or sexual selection and which ones cannot. In this process, as long as they are all grounded in natural selection, distinct theories may be easier to subsume under an overarching theory (Savage et al., 2021), but as soon as sexual and natural selection mechanisms both play a role, theory building becomes more challenging (Keller et al., 2023). Another related challenge will be to develop explanatory theories that describe the specific evolutionary mechanisms involved in the emergence of musicality, along with their respective time-scales (see also Novaes and Natividade, 2023).

To be logically consistent with evolutionary theory, adaptive accounts require evidence of the heritability of at least some components of musicality, as well as evidence of the presence of a selectively acquired function (Justus and Hutsler, 2005; McDermott and Hauser, 2005; Croston et al., 2015). While it is difficult to assess the selective pressures faced by our ancestors with respect to musicality (Honing et al., 2015), studies have shown a large phenotypic variability in musicality among humans (Müllensiefen et al., 2014), which is at least partly heritable (Gingras et al., 2015a; Mosing et al., 2015; Wesseldijk et al., 2023). Thus, currently available evidence suggests that adaptationist accounts of the origins of musicality have cleared this initial hurdle, although much work remains to be done.

With respect to Darwin’s sexual selection hypothesis, Mosing et al. (2015) reported that, although some components of musicality, such as pitch, melody, and rhythm perception (as measured by test batteries, see Ullén et al., 2014), were moderately heritable in a sample of over 10,000 Swedish twins, musical ability and mating success were negatively associated. Moreover, Harrison and Hughes (2017) reported similar sexual activity for musicians and non-musicians (but see Lange and Euler, 2014). However, recent experimental studies have provided support for Darwin’s hypothesis (see Section 4).

## 3 Music-induced emotions

The study of emotions expressed or induced by music has a long history (e.g., Juslin and Sloboda, 2011). Music has repeatedly been shown to induce the expressive, psychophysiological and subjective feeling components of an emotion episode (Krumhansl, 1997; Ogg et al., 2017; Fuentes-Sanchez et al., 2021), as proposed by Scherer (2009) in his component process model of emotion. Indeed, music can induce intense pleasure and chills (Blood and Zatorre, 2001; Grewe et al., 2007) and emotional lacrimation (Wassiliwizky et al., 2017; Mori and Iwanaga, 2021). These strong pleasurable responses are mostly due to music’s ability to activate the human reward system (Ferreri et al., 2019; Fasano et al., 2023) and to induce emotional arousal responses in listeners (Schafer and Sedlmeier, 2011; Gingras et al., 2015b). Arousal has also been found to be a correlate of perceived musical complexity, which is central to Berlyne’s psychobiological model of aesthetic responses (Berlyne, 1960; Marin, 2022). Another key finding is that similar acoustical cues convey emotion communication in speech and music (Juslin and Laukka, 2003), which supports the hypothesis of a common origin of music and speech (see Section 2).

The psychological mechanisms through which music induces emotions are manifold and involve various brain functions. They can be described as brain stem reflex, rhythmic entrainment, emotional contagion, musical expectancy, evaluative conditioning, visual imagery, episodic memory and aesthetic judgment (Juslin, 2013). Each mechanism is hypothesized to have a specific adaptive value and to be associated with specific types of affective responses. Music has been documented to induce emotions and moods in various social and situational contexts, ranging from live music concerts to self-selected music listening in private and public spaces (Sloboda and O'Neill, 2001; Juslin and Laukka, 2004; North and Hargreaves, 2008). In these various contexts, music can consciously or unconsciously influence people’s feelings and behavior (North and Hargreaves, 2008). For example, background music can affect eating behavior as well as time and money spent in restaurants (Stroebele and de Castro, 2006; Beer and Greitemeyer, 2019). Film music is a prominent example of how the processing of visual information can be altered by musical emotions (Steffens, 2020; Herget, 2021), and musical arousal even impacts driving behavior (van der Zwaag et al., 2013; Navarro et al., 2019). Music listening is also an excellent means of regulating one’s emotions in a wide range of everyday situations outside the therapeutic context (Cook et al., 2019; Bachman et al., 2022; Garrido et al., 2022). Positive effects of emotion regulation by music have also been reported in the context of child care, parent-infant communication and social bonding (Persico et al., 2017; Cirelli et al., 2020; Whittall et al., 2023).

## 4 Musicality and its role in sexual attraction

Following Miller’s (2001) revival of Darwin’s sexual selection hypothesis, Charlton et al. (2012) and Charlton (2014) experimentally tested the idea that females may be more sensitive to musical cues signalling genetic quality during peak fertility (Gangestad and Thornhill, 2008). The 2012 study used simple computer-generated melodies that varied in complexity. One group of females rated the complexity of the musical stimuli, whereas a second group gave liking ratings for the same stimuli at two points during the menstrual cycle (fertile versus infertile). The results did not reveal a significant effect of conception risk on liking ratings. In Charlton’s (2014) follow-up study, one group of female participants listened to pairs of laboratory-generated musical excerpts, allegedly from different composers, and was asked to choose which composer sounded the most complex. A second group was asked to indicate which composer they preferred as a sexual partner in either a short-term or a committed long-term relationship. Conception risk affected preference ratings for a short-term relationship, with females in the fertile phase of their cycle preferring composers of complex melodies, but not for a long-term one. These findings suggest that musical creativity could be an indicator of “good genes” and were interpreted as evidence for sexual selection (see also Miller, 2001; Haselton and Miller, 2006; Varella et al., 2022).

Although Charlton’s studies did not test males, they introduced elegant experimental paradigms demonstrating that musical creativity and complexity may influence female mate choice. However, because complexity is associated with higher arousal (see Section 3), it is unclear whether participants based their decisions purely on the quality of the musical compositions and their complexity (as an indicator of creativity) or whether the reported effects may be explained by arousal induction. Moreover, Charlton focused on the effects of musical sound in isolation, without accounting for other potential mating cues.

To circumvent these limitations, Marin et al. (2017) and Marin and Rathgeber (2022) developed a crossmodal priming paradigm to examine whether music and musicality affect sexual attraction in females and males. This research strand builds upon a body of studies suggesting that music-induced emotions can alter visually-induced emotions (e.g., Jeong et al., 2011), with arousal and pleasantness showing differential effects (Marin et al., 2012; Lee et al., 2017). Moreover, Marin et al. (2017) and Marin and Rathgeber (2022) employed facial targets because facial attractiveness is an important biological cue in mate choice (Currie and Little, 2009), in which overall sexual attraction is determined by a complex interplay of several physical, cognitive and social cues (Buss and Schmitt, 2019).

Misattribution of arousal (i.e., excitation transfer, Zillmann, 1983, see also Dutton and Aron, 1974; Foster et al., 1998) was identified by Marin et al. (2017) as a potential psychological mechanism through which music can affect mate choice (see also May and Hamilton, 1980). Musical primes varying in arousal and pleasantness were selected from the 19th-century piano repertoire. High-arousing musical excerpts were also more complex than low-arousing excerpts. Compared to a silent control condition, females gave higher facial attractiveness and dating desirability ratings in response to opposite-sex faces after musical priming, with high-arousing excerpts showing the largest effects. Conception risk did not significantly affect these ratings (but see Charlton, 2014). There were no significant effects in males. In their follow-up study, Marin and Rathgeber (2022) focused on musicality and modified the instructions accordingly. Participants were told that the musical excerpts were performed by the people shown on the photographs. A significant increase in facial attractiveness and dating desirability after musical priming was found in females, but no specific arousal/complexity effect. In males, dating desirability (but not attractiveness) ratings increased after musical priming. Ideally, studies on music and sexual selection should include only singles (as in Marin and Rathgeber, 2022, but unlike Marin et al., 2017) and only females that are not using hormonal contraception (as in both studies led by Marin).

Marin et al. (2017) and Marin and Rathgeber (2022) offered some insights into the psychological mechanisms by which instrumental music may affect sexual attraction in listeners. In both studies, a crossmodal priming paradigm was employed with the same stimulus materials but different instructions. One can thus conclude that excitation-transfer effects are clearly observable when musical primes and facial targets are not explicitly linked by the instructions, but not when primes and targets are linked. This suggests two possible (partly interwoven?) pathways by which music(ality) may affect partner choice: one route that is rather affect-based, and another that showcases the musician as someone having advanced motoric and expressive skills (Miller, 2000). Since arousal (affective route) and complexity (honest signal) are often interrelated in music (see Marin and Leder, 2013), further studies using different musical styles and experimental paradigms are necessary. Studies should also incorporate a wider range of physical mate cues (Groyecka et al., 2017), such as the human voice, and examine performer-listener interactions given recent observations suggesting intrasexual competition in human chorusing (Keller et al., 2023). Overall, Marin’s findings are in agreement with Darwin’s (1871, p. 880) claim that “the progenitors of man, either the males or females or both sexes, […] endeavoured to charm each other with musical notes and rhythm”.

Focusing on musical creativity as a fitness indicator, Madison et al. (2018) studied multiple cues in mate choice and combined faces of three levels of attractiveness with musical excerpts of three levels of performance quality. The authors framed their research in the context of Darwin’s sexual selection hypothesis and parental investment theory (Trivers, 1996). They tested female and male participants who looked at an opposite-sex face while listening to musical improvisations which were all played at the same tempo and in major mode. This minimized affective differences between the performance quality levels and thus facilitated the interpretability of the effects. Participants were asked to rate mate value (intelligence, health, social status and parenting skill) and mate preference scales (date, intercourse, short- and long-term relationship) as well as attractiveness. In general, improvisation skills increased all types of ratings with a few exceptions; however, facial attractiveness had a much larger effect than performance quality, and exerted a smaller influence on ratings among females than among males. Altogether, the results do not falsify the sexual selection hypothesis of music evolution.

Darwin (1871, p. 332) observed that in “most of the lower classes [non-mammals] the sounds produced by the males, serve not only to call but to excite or allure the female”. At that time, evidence for Darwin’s observation was lacking among mammals. Kreutz (1997) hypothesized that music may affect erotic relationships by intensifying perceptions and emotions in humans. Indeed, there is now empirical evidence for music’s role in sexual arousal (Wan and Lalumière, 2017; van Bohemen et al., 2018; Tikka et al., 2022; but see Grewe et al., 2009) and sexual fantasies (Lehmiller, 2018). These findings may be interpreted as supporting the view that sexual selection plays a role in the evolution of musicality. In this context, one may be inclined to take the existence of love songs and its role in romantic pair bonding (Dukes et al., 2003; Hobbs and Gallup, 2011) as further evidence for sexual selection. However, romantic relationships (and pair bonding) between men and women, as we understand it nowadays, presumably did not exist in hunter-gatherer tribes and were probably not expressed in songs. Monogamous relationships only became the dominant model late in our history (perhaps around 10,000 years ago) when the development of agriculture considerably changed mating behavior (see Miller, 1998). Moreover, Brown (2000b) has argued that interactions between music and language, such as in songs with lyrics, appeared after music and language evolved as two distinct communication systems out of one common hominid referential emotive vocalization system. Therefore, we consider the study of love songs to be secondary when trying to address the possible roots of human musicality.

Other supportive evidence for music’s role in sexual attraction stems from simulation studies (Werner and Todd, 1997; Van den Broek and Todd, 2009), studies investigating the influence of depicting musical instruments on men’s profile pictures in social media (Tifferet et al., 2012, but see Wassiliwizky et al., 2023) and the role of music and musicianship in self-descriptions in the context of online dating (Lee et al., 2019). However, Bongard et al. (2019) reported that verbal profiles of musicians were not rated as more attractive than those of non-musicians, but raters who were musically interested found musicians attractive in private settings (see also Montoya et al., 2008). Chang et al. (2021) studied the effect of body sway in a speed dating paradigm with groovy background music and found that, compared to low-groove music, high-groove music led to an increased interest in meeting the partner again, suggesting that social bonding mechanisms based on groove and entrainment may be another route through which music may increase sexual attraction (see Savage et al., 2021). Interestingly, groovy music has been associated with heightened arousal (Bowling et al., 2019), implying a potential arousal-based mechanism. Another recent study suggests that the practice behavior of heavy metal guitarists may be related to status seeking and mate attraction (DeLecce et al., 2022).

## 5 Discussion

This review has shown that empirical evidence for music’s role in sexual attraction has accumulated in the last decade, mostly stemming from behavioral studies focusing on listeners’ perceptions and subjective responses. First, musicality, including mere visual indication of musical proficiency (Tifferet et al., 2012; Lee et al., 2019), has been demonstrated to be an attractive trait, especially among female listeners (Charlton, 2014; Madison et al., 2018; Marin and Rathgeber, 2022). Second, the emotional impact of music seems to enhance perceived attractiveness (Marin et al., 2017; Chang et al., 2021) and sexual arousal (Tikka et al., 2022) in listeners. However, a fully convincing argument for sexual selection based on behavioral measures would require demonstrating greater mating success among musically skilled individuals (Ravignani, 2018; see also Section 2), or at least, more frequent (and perhaps risky) sexual behavior. On the other hand, the role of sexual selection will only be completely refuted if the best available evidence (for instance, obtained from a cross-cultural meta-analysis) refutes every plausible sub-mechanism of sexual selection in both performers and listeners. It is possible that only a few mechanisms of sexual selection have significant explanatory power in the evolution of musicality.

The issue of sexual dimorphism in relation to sexual selection warrants further research (Varella et al., 2010, 2017, 2022). For instance, studies employing both female and male participants and depictions of opposite-sex faces have shown that males’ ratings of female facial attractiveness are nearly impervious to music or cues of musicality, whereas females appear to be more sensitive to such cues (Marin et al., 2017; Madison et al., 2018; Marin and Rathgeber, 2022; see also Kaufman et al., 2016). The mutual mate choice model, which argues that sexual dimorphism is low among humans, although some sex differences remain, may help explain these results (Miller, 2013). Thus, the absence of significant sex differences in basic music perception skills (Bertolo et al., 2023) does not provide convincing evidence against sexual selection, since both sexes need to be able to perceive and evaluate aesthetic displays (Miller, 2001; Varella, 2023). In general, the current empirical evidence seems to be in line with Darwin (1871), who did not regard music as a sexual dimorphic trait.

Future challenges include the study of other facets of musicality besides instrumental music, such as singing (Grewe et al., 2009; Valentova et al., 2019; Keller et al., 2023), dance (Weege et al., 2015; Garfinkel, 2018; Fink et al., 2021), and beat synchronization (Ravignani, 2018). Different sexual selection mechanisms may have shaped these various aspects of musicality to different degrees. In this regard, it may be fruitful to work closely together with ethnomusicologists to collect meaningful cross-cultural data in laboratories as well as in the field. A strictly Western perspective will not be sufficient when studying the origins of music.

Another strand of research should explore in greater depth the underlying mechanisms by which music and musicality affect mate choice, with a particular emphasis on music-induced emotions. In the long term, this will offer insights into how affect and cognition interact in decision-making processes related to sexual behavior, which will be of interest to fields outside music psychology. At the moment, affective arousal appears to be the key player explaining how music affects sexual attraction. Arousal-based theories such as “misattribution of arousal” (Schachter and Singer, 1962), “excitation-transfer” (Zillmann, 1983) or the “arousal-as-information” framework (Storbeck and Clore, 2008) are helpful to explain some of the observed effects. Motivational theories, such as Berlyne’s (1960) psychobiological model, which is still frequently cited in the field of empirical aesthetics (Marin, 2022), also consider arousal as vital in determining hedonic responses to different kinds of artworks including music. Nevertheless, the role of valence and more complex emotions (Clore, 1992; Angie et al., 2011; DeWall et al., 2016) in social judgments and decision-making regarding musicality and sexual attraction has yet to be explored in depth. When studying these decision-making processes during music listening, it will also be important to disentangle effects that are based on evaluations of musical abilities (performance quality or musical creativity) from emotion-based influences on sexual attraction.

Furthermore, the impact of musicality and music on different types of mate preferences (Buss and Schmitt, 2019) has not been sufficiently studied so far (but see Charlton, 2014; Madison et al., 2018). Although short-term mating behavior is most relevant for the theory of sexual selection, music activities may increase the quality of long-term relationships by creating intimacy between partners (see Kreutz and Feldhaus, 2023). Moreover, a broader spectrum of mate cues needs to be studied to get a better understanding of the relative importance of music-related cues, as well as other cues of human artisticality (Varella, 2021), creativity (Kaufman et al., 2016; Novaes and Natividade, 2023) and humor (Kaufman et al., 2008). Finally, future studies should account for cultural stereotypes related to musical styles (Zillmann and Azra, 1989), and consider music preferences and different types of musical proficiency in their research designs (Bongard et al., 2019).

## Author contributions

MM: Conceptualization, Writing – review & editing, Writing – original draft. BG: Conceptualization, Writing – original draft, Writing – review & editing.
